# A Case of Renal Iron Overload Associated with Cold Agglutinin Disease Successfully Managed by Rituximab

**DOI:** 10.46989/001c.91478

**Published:** 2023-12-29

**Authors:** Kathryn Taberner, Andrew A. House, Aaron Haig, Cyrus C. Hsia

**Affiliations:** 1 Department of Medicine London Health Sciences Centre; 2 Department of Medicine, Division of Nephrology London Health Sciences Centre; 3 Department of Pathology and Laboratory Medicine London Health Sciences Centre; 4 Department of Medicine, Division of Hematology London Health Sciences Centre

**Keywords:** Cold agglutinin disease, hemolysis, renal hemosiderosis, rituximab, iron overload

## Introduction

Renal hemosiderosis is a disease in which iron or hemosiderin deposits in the renal cortex as a form of iron overload.^1^ Renal hemosiderosis in the context of severe intravascular haemolysis is typically associated with paroxysmal nocturnal haematuria (PNH).^2,3^ There have been cases of renal hemosiderosis reported with sickle cell anaemia, hemochromatosis, and prosthetic heart valves.^4,5^ There has been one report of renal hemosiderosis occurring with cold agglutinin disease, but this was associated with heavy alcohol intake.^6^ We report a patient with cold agglutinin disease (CAD) who developed renal hemosiderosis due to severe intravascular haemolysis which was successfully managed with rituximab.

## Case Report

A 71 year old woman presented with several months of increasing fatigue and tea coloured urine. Relevant past medical history included type-2 diabetes mellitus managed with diet, and hypertension on candesartan. Historically, she consumed as many as 14 alcoholic beverages per week, but this was over a period of approximately one year in her forties, and she had substantially decreased her intake in recent years to about 2 drinks per week. There was no history of recent infection, medication changes, travel, or any symptoms of connective tissue disease. Further, there was no known personal or family history of hemochromatosis. On examination, the patient had normal vital signs, normal cardiovascular and respiratory exams, a palpable liver 2-3 cm below the costal margin, with jaundice and scleral icterus. Laboratory testing demonstrated a haemoglobin (Hb) of 96 g/L, mean corpuscular volume (MCV) 98.3 fL, and platelets of 274 x 10^9^/L. Reticulocytes were 154 x 10^9^/L, with a lactate dehydrogenase (LDH) of 595 U/L, haptoglobin of less than 0.10 g/L, total bilirubin of 51 µmol/L (direct bilirubin of 11 µmol/L) (Table 1). Microscopic examination of the peripheral blood smear demonstrated red blood cell agglutination. Direct antiglobulin test (DAT) was positive, demonstrating C3 coating the red blood cells. Cold agglutinin screen was positive, cold agglutinin titre was 1 in 128, and a thermal amplitude was 30°C. Human immunodeficiency virus (HIV) and Hepatitis B testing were negative, and an abdominal ultrasound showed a normal liver and spleen. Glucose-6-phosphate dehydrogenase (G6PD) activity was normal at 16.5 U/g Hb. Iron indices showed a serum iron 45 µmol/L (6-27), total iron binding capacity 56 µmol/L (45-77), transferrin saturation 0.80 (0.20-0.50) and ferritin of 214 mcg/L (normal 12-287). Molecular genetic testing for *HFE* gene mutations was not available.

**Table 1.** Lab Values at Initial Clinic Visit and After Completion of Rituximab Course

Lab Values	Initial Visit	Post rituximab (~1 month)
White Cell Count (4.0-10.0 x 10^9^/L)	7.7	8.5
Haemoglobin (115-160 g/L)	96	117
MCV (80-100 fL)	98.3	94.1
Platelets (150-400 x 10^9^/L)	274	243
Reticulocytes (10-100 x 10^9^/L)	154	104
LDH (less than 214 U/L)	595	369
Total Bilirubin (<21 µmol/L)	51	30
Haptoglobin (0.30 - 2.00 g/L)	< 0.10	<0.10
C3 (0.90-1.80 g/L)	1.03	1.16
C4 (0.10-0.40 g/L)	0.09	0.13
C ANCA (<19 RU/mL)	17	16
P-ANCA (<19 RU/mL)	47	60
anti GBM (<19 RU/mL)	40	34
Creatinine (55-100 µmol/L)	56	55
Albumin/creatinine ratio (mg/mmol)	50.3	29.0

She was assessed by a nephrologist given the presence of proteinuria (3+) and haematuria on urine dipstick. Under microscopy, the urine was deeply pigmented, likely due to bilirubin and haemoglobin, but had very few cells and no cellular casts. The urine albumin to creatinine ratio was elevated at 50 mg/mmol creatinine but serum creatinine was normal at 55 µmol/L, with estimated glomerular filtration rate (CKD-EPI 2021 formula) of 95 mL/min/1.73m^2^ . Rheumatoid factor, lipids, glycated haemoglobin, cytoplasmic antineutrophil cytoplasmic antibodies (C ANCA), and complement component 3 (C3) were all normal. Perinuclear antineutrophil cytoplasmic antibodies (P ANCA) and anti-glomerular basement membrane antibodies (anti-GBM) were both positive (47 and 40 RU/mL, respectively) with a decreased complement component 4 (C4) (0.09 g/L) (Table 1). A renal biopsy revealed 18 glomeruli, with 1 globally sclerotic glomerulus. By light microscopy, the tubular epithelium contained abundant hemosiderin pigment, as confirmed with Perl’s Prussian blue histochemical staining. Congo red staining was negative for amyloid. Immunofluorescent labelling was negative for IgA, IgG, IgM, kappa, lambda, and fibrinogen (Figure 1). Electron microscopy showed no significant abnormalities of the basement membrane or effacement of the foot processes. Ultimately, the kidney biopsy revealed renal tubular hemosiderosis, with no significant chronic changes or features of vasculitis or rapidly progressive or acute glomerulonephritis.

**Figure 1a.**
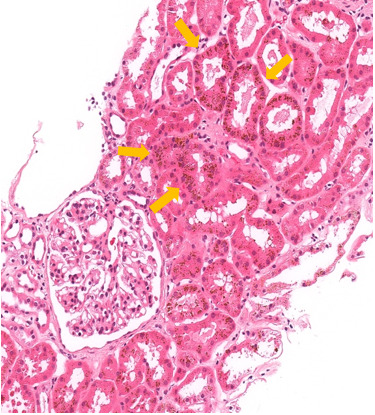
Hematoxylin and eosin staining shows brown pigment deposition in the proximal tubular epithelial cells (arrows).

**Figure 1b.**
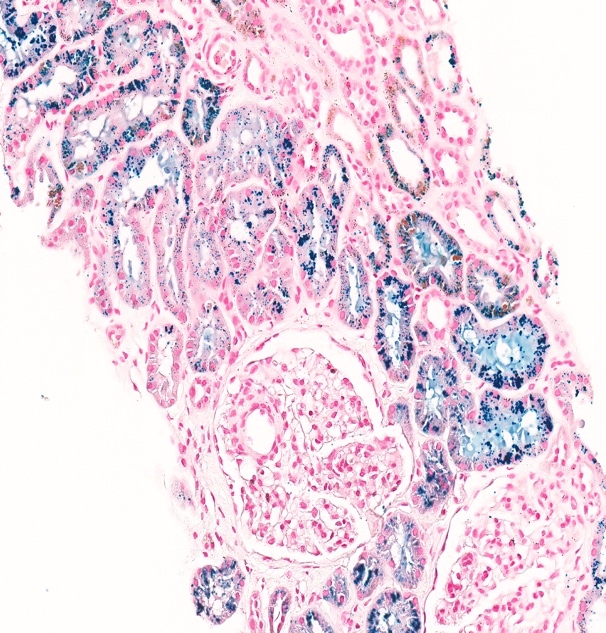
Perl’s Prussian blue histochemical staining highlights iron deposition in the tubular epithelium (blue staining).

The patient was initiated on 5 mg of folic acid and instructed to avoid the cold. It was ultimately decided to initiate infusions of rituximab (riximyo) 375mg/m^2^ weekly for a total of 4 weeks. The patient’s symptoms improved after completion of the 4 sessions of rituximab. Hb and reticulocyte count normalized, while total bilirubin (28 µmol/L) and LDH (310 U/L) improved but remained somewhat elevated, with a haptoglobin less than 0.10 g/L, consistent with ongoing low-grade compensated haemolysis. Repeat anti-GBM and P-ANCA levels were assessed and remained elevated (34 and 60 RU/mL) after treatment with rituximab, but were not felt to be clinically significant.

## Discussion

CAD accounts for 15% of autoimmune haemolytic anaemias, and is characterized by agglutination of red blood cells at temperatures below the normal core body temperature.^7,8^ It affects more females than males, typically in their seventies.^9^ Cold agglutinin disease can be divided into primary and secondary, with the latter being associated with autoimmune disorders, viral infections, and haematological malignancies.^10^ The exact underlying cause of this patient’s CAD was undetermined and may be idiopathic. She had no preceding viral illness and no findings to indicate an underlying malignancy. The patient was found to have elevated levels of P-ANCA and anti-GBM before and after the use of rituximab, although she did not have any specific clinical features associated with these respective diseases, and renal biopsy was not consistent with a diagnosis of ANCA-associated vasculitis or anti-GBM disease. Patients with autoimmune diseases (CAD) can have other autoantibodies that may not be clinically significant.

Patients with CAD typically present with clinical features of mild acrocyanosis and disabling Raynaud’s phenomenon.^10^ These cold induced symptoms are present in about 90% of patients.^11^ Our patient’s primary complaint was fatigue, along with slight jaundice and scleral icterus. The average Hb in those with CAD is around 90 g/L, and the median total bilirubin is 41 µmol/L.^11^ Upon presentation to our clinic, our patient’s Hb was 96 g/L, but was found on previous tests to be as low as 83 g/L with a total bilirubin of 51 µmol/L. CAD is associated with relatively mild haemolysis, in contrast to more severe hemolysis in PNH, which is why the finding of renal hemosiderosis is more commonly associated with PNH, and not CAD. Haemoglobinuria also tends to be more severe in PNH in comparison to CAD.^12^ In the present patient, CAD was associated with severe haemolysis resulting in elevated haemolysis markers and renal hemosiderosis. There has been one other report of renal hemosiderosis occurring with CAD, but this was associated with heavy alcohol intake, which could have contributed to the degree of hemolysis. Our patient did have a history of alcohol use, but she significantly decreased her alcohol intake prior to presentation, and also had normal liver function tests.

The sequelae of intravascular haemolysis causing renal hemosiderosis is as follows. During haemolysis, haemoglobin escapes into the plasma, dimerizes and is bound to haptoglobin. In the setting of prolonged or massive haemolysis, haptoglobin becomes depleted. Hb unbound to haptoglobin is filtered by the glomerulus and absorbed in the proximal convoluted tubules where it is catabolized with the release of iron in the form of hemosiderin.^13^ Repeated exposure of the renal epithelium to Hb leads to progressive intense hemosiderin deposition in the proximal convoluted tubule,^14^ which was evident on biopsy in our patient’s case. This deposition can lead to tubular damage and, eventually, to an acute kidney injury. Our patient presented with mild proteinuria and haematuria, but her creatinine and renal function remained normal throughout her clinical course. The mechanisms by which renal hemosiderosis leads to acute kidney injury can include direct cytotoxic effects, decreased renal perfusion due to depletion of nitric oxide, and cast nephropathy.^6^

Our patient was successfully treated with rituximab, which is considered to be the standard therapy for CAD.^15^ She was also educated to avoid the cold, and to continue on folate replacement indefinitely. Sustained remission is unlikely, with 57-89% of responsive patients eventually relapsing, with median response duration of 11 months.^12^ However, rituximab has been successful in treating relapsed CAD. Her hemolytic anaemia was successfully managed with rituximab; however, it remains unclear if it improved the renal hemosiderosis. Her renal function remained at baseline, with a creatinine in the 50s throughout, and improvement in her microalbumin/creatinine ratio. A repeated renal biopsy could be contemplated to identify any improvement in her renal hemosiderosis. However, there are risks associated with such a procedure, so a repeat biopsy has been deferred, unless it may lead to a change in management. The utilization of an iron magnetic resonance imaging may have been an alternative option to assess iron overload status, but it was not performed in this case.

In summary, our case uniquely demonstrates the development of renal hemosiderosis secondary to haemolysis in the setting of CAD with response to rituximab.

### Credit authorship contribution statement

Data curation: Kathryn Taberner (Equal), Aaron Haig (Equal). Formal Analysis: Kathryn Taberner (Lead). Writing – original draft: Kathryn Taberner (Lead). Writing – review & editing: Kathryn Taberner (Equal), Andrew A. House (Equal), Aaron Haig (Equal), Cyrus C. Hsia (Equal). Conceptualization: Andrew A. House (Equal), Cyrus C. Hsia (Equal). Supervision: Cyrus C. Hsia (Lead). Validation: Cyrus C. Hsia (Lead).

### Competing Interests

The authors have no competing interests to declare that are relevant to the content of this article.

### Informed Consent

The participant has consented to the submission of the case report to the journal.

### Availability of Data

Data available upon request to corresponding author.
